# Construction of an evaluation index system for user satisfaction with immersive virtual reality exergaming

**DOI:** 10.3389/fpsyg.2026.1811648

**Published:** 2026-04-17

**Authors:** Jian Fu, Nanyue Mou, Duoyou Gong, Youzhi Xu, Xiaoning Lu

**Affiliations:** 1 College of Physical Education and Health, Guangxi Normal University, Guilin, Guangxi, China; 2 Guangxi College of Sports Education, Nanning, Guangxi, China; 3 Guilin University, Guilin, Guangxi, China; 4 Tongmyong University, Busan, Republic of Korea; 5 Silla University, Busan, Republic of Korea

**Keywords:** evaluation index system, G1-CRITIC, grounded theory, immersive virtual reality exergaming, user satisfaction, VIKOR

## Abstract

Advances in virtual reality technology have given rise to immersive virtual reality exergaming (IVRE), offering new opportunities to increase public physical activity levels and promote the development of recreational sports. This study proposes a systematic evaluation framework for user satisfaction (USAT) during IVRE usage, providing measurement instruments and decision-making support for assessing and improving USAT. We first used a qualitative method called grounded theory to analyze in-depth interviews with users. This helped us identify the key factors that shape their satisfaction. Then, we combined expert opinions with a statistical technique (the G1-CRITIC method) to determine how important each of these factors is. Finally, we tested our framework on eight popular IVRE platforms using a multi-criteria decision-making tool (the VIKOR method) to confirm that it works in practice. The results indicate that the developed USAT evaluation framework comprises five primary indices, fourteen secondary indices, and fifty-seven tertiary indices. Empirical findings demonstrate that the framework exhibits satisfactory reliability and validity. This study provides a methodological and empirical foundation for the design and development of IVRE, with an emphasis on enhancing user experience. Moreover, it extends existing theoretical perspectives and methodological approaches in IVRE research, offering a valuable reference for future related studies.

## 1 Introduction

Immersive Virtual Reality Exergaming (IVRE) has attracted increasing scholarly and industrial attention as an emerging form of physical activity, owing to its high level of embodied interaction, low spatial constraints, and favorable cost efficiency (Lee and Kim, 2018). However, despite the growing availability of IVRE products, user acceptance remains uneven. User satisfaction (USAT) plays a pivotal role in enhancing user experience, fostering sustained usage intention, and supporting long-term product viability (Hu et al., 2023). Accordingly, the scientific evaluation of USAT throughout the design and development stages of IVRE is critical.

Existing research on IVRE has predominantly focused on intervention effectiveness, target user groups, and product development pathways (Cao et al., 2021; Grosprêtre et al., 2023; Martin-Niedecken and Mekler, 2018; Plante et al., 2003), while systematic investigations into USAT evaluation index systems for IVRE remain scarce. According to GLOBE NEWSWIRE, the immersive virtual reality market is projected to reach US$120.74 billion by 2032 (https://www.snsinsider.com/sample-request/5987), suggesting substantial growth potential for IVRE. As USAT constitutes a key indicator of user acceptance (Hu et al., 2023; Liu, 2024), well-defined evaluation indices are essential for accurately assessing user experience and guiding sustainable product development (Fang et al., 2025; Zhang and Ni, 2023). Therefore, the development of a comprehensive, scientific, and standardized USAT evaluation system for IVRE is both necessary and timely.

Our research approach is threefold. First, we will use grounded theory to systematically explore user experiences and build a preliminary list of satisfaction indicators. Second, we will apply the G1-CRITIC method—a combined approach that balances expert judgment with objective data—to assign weights to these indicators, showing which factors matter most. Third, we will use the VIKOR method to evaluate eight existing IVRE platforms. This final step will test our proposed framework, thereby validating the effectiveness and applicability of the proposed evaluation indicator system.

## 2 Relevant research

### 2.1 IVRE user satisfaction: a nascent research area

Existing research on IVRE has predominantly focused on its intervention effectiveness (e.g., improving physical function, cognitive enhancement) and technical development (Ko et al., 2020; Ren et al., 2023; Shah et al., 2023). While these studies establish IVRE's potential as a beneficial tool, they offer limited insight into the user experience itself. Studies on user experience in IVRE, though growing, often examine specific features like feedback mechanisms or social presence (Xu et al., 2023). A holistic, theoretically grounded understanding of what constitutes USAT in this context remains underdeveloped. This gap is significant because, as Hu et al. (2023) note, USAT is a key driver of user loyalty and long-term engagement, which are critical for the sustainable success of any interactive technology. Therefore, a systematic investigation into the multi-dimensional nature of USAT in IVRE is both timely and necessary.

### 2.2 Current research on evaluation index systems

Constructing a robust evaluation framework involves three core tasks: identifying relevant indicators, determining their relative importance (weighting), and validating the framework (Yang et al., 2023; Qi et al., 2024).

(1) Identifying Indicators. Previous studies have built evaluation frameworks using literature reviews (Giovanelli et al., 2015; Liu, 2024) or expert interviews (Fang et al., 2025; Shen et al., 2014). While valuable, these top-down approaches risk missing factors that are salient to end-users but not yet documented in the literature or fully recognized by experts. In contrast, grounded theory offers a powerful bottom-up alternative. As a qualitative methodology, it is designed to generate theory directly from the perspectives and experiences of participants, making it particularly suitable for exploring new or under-theorized domains (Qi et al., 2024; Sbaraini et al., 2011; Turner and Astin, 2021; Yang et al., 2023). Given the nascent state of USAT research in IVRE, we adopt grounded theory to ensure our indicator system is comprehensively grounded in authentic user experiences.
(2) Weighting Indicators. To prioritize the identified factors, we must assign them weights. Subjective methods like the G1 method and the Analytic Hierarchy Process (AHP) rely on expert judgment to determine importance (Li et al., 2023a; Tuskan and Basari, 2023). While this captures domain expertise, it can be influenced by individual bias. Conversely, objective methods like CRITIC derive weights purely from the statistical properties of data (e.g., variability and correlation among indicators; Krishnan et al., 2021). This approach is data-driven but may lack the contextual understanding that experts provide. Acknowledging the limitations of relying on a single perspective, recent research has advocated for combined weighting methods (Lu et al., 2024; Qi et al., 2024; Zhao et al., 2021). The G1-CRITIC method integrates the strengths of both approaches: it balances the informed opinions of experts with the empirical evidence from user data, leading to a more balanced, reliable, and theoretically sound set of indicator weights (Ali, 2022; Krishnan et al., 2021; Li et al., 2023b; Wang et al., 2017; Ye et al., 2023; Zhang et al., 2023).
(3) Validating the Framework. Finally, the proposed framework must be tested. Multi-criteria decision-making methods are well-suited for this task, as they can rank multiple alternatives based on a set of weighted criteria (Sun et al., 2023; Tuskan and Basari, 2023; ul Amin et al., 2022). Among these, the VIKOR method is particularly useful for problems with conflicting criteria, as it determines a compromise solution by considering both the “group utility” (average performance) and “individual regret” (the worst-performing criterion) of each alternative (Kun et al., 2018; Li et al., 2022; Tuskan and Basari, 2023). This characteristic makes VIKOR ideal for evaluating IVRE platforms, where a product might excel in some areas but lag in others. By applying VIKOR, we can not only rank existing IVREs but also rigorously test the discriminative power and practical utility of our newly constructed USAT evaluation system.

### 2.3 Methodology

**Figure 1:**
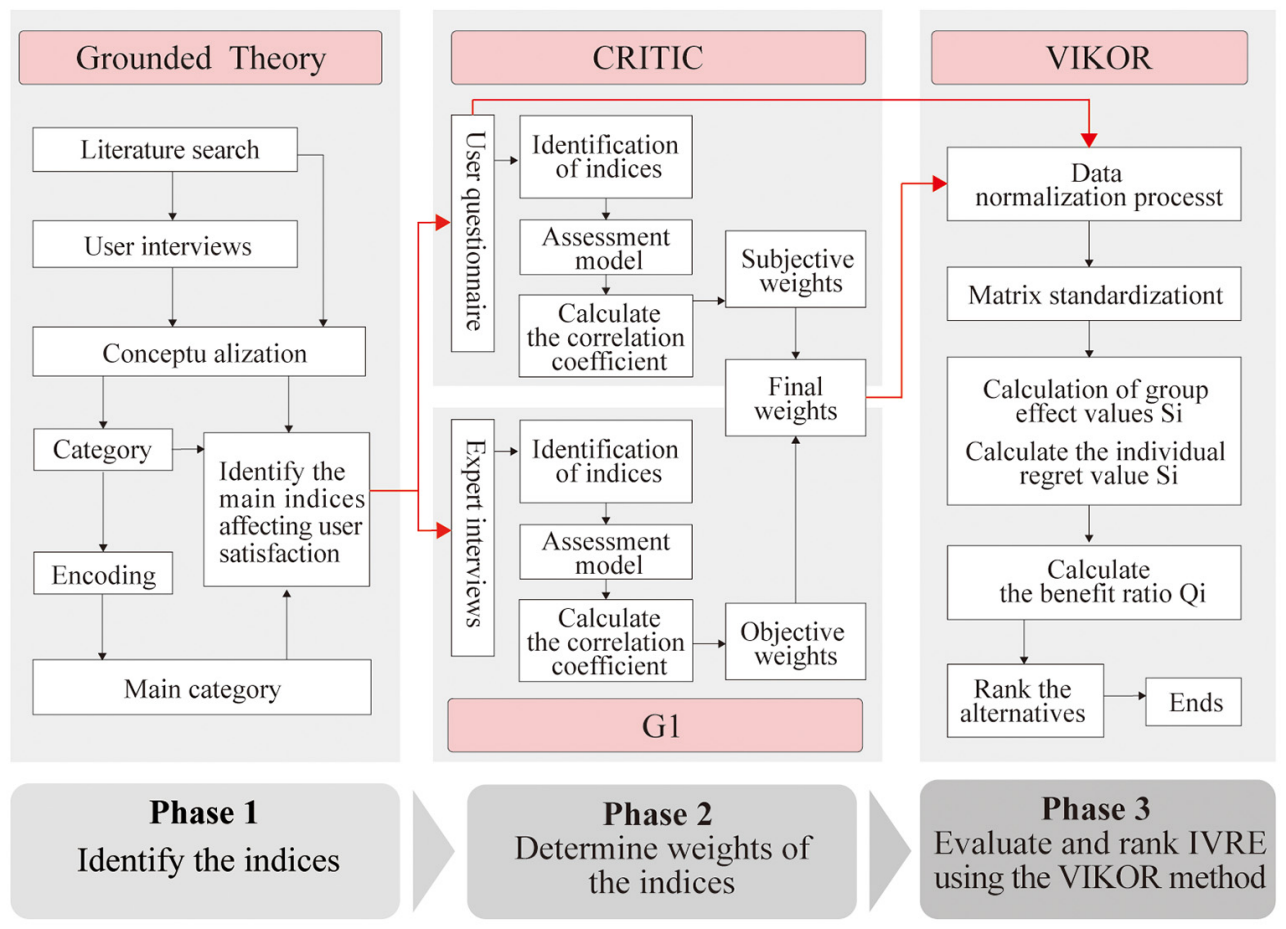
Research process.

This study is divided into three main stages, as illustrated in Figure 1.

Stage 1: identifying the Indicators. We used grounded theory, a rigorous qualitative method, to analyze interview transcripts. This process (open, axial, and selective coding) allowed us to systematically derive a comprehensive list of satisfaction indicators directly from user experiences.

Stage 2: weighting the Indicators. We then needed to determine the relative importance of these indicators. To do this objectively and reliably, we used a combined weighting method. We asked experts to rank the indicators (the G1 method for subjective weights) and also analyzed user questionnaire data to see how much opinions varied on each point (the CRITIC method for objective weights). We then mathematically combined these two sets of weights (Zhao et al., 2021).

Stage 3: validating the Framework. Finally, we tested our complete framework by using it to evaluate eight popular IVRE products. We used the VIKOR method, which is designed to rank options based on multiple criteria, to see if our framework's rankings matched up with what users actually thought.

## 3 Determining evaluation indices for USAT

### 3.1 Data collection

This study employed semi-structured interviews to collect user data. Guided by an interview protocol, researchers conducted open-ended, interactive discussions to obtain rich information (Dolczewski, 2022), thereby capturing participants' authentic perspectives and experiential insights in depth (Hu et al., 2023; Kallio et al., 2016). One-on-one interview sessions were adopted to enhance data quality and ensure focused communication (Doody and Noonan, 2013).

#### 3.1.1 Interview protocol and procedures

The semi-structured interview protocol was developed based on a preliminary review of the IVRE literature and discussions among the research team. The protocol covered four main thematic areas: (1) Overall experience with IVRE: participants were asked about their general experiences using IVRE, including what motivated them to start, how frequently they use it, and what they perceive as the main benefits and drawbacks. (2) Specific aspects of the experience: questions explored participants' perceptions of immersion, usability, interactivity and enjoyment. (3) Social dimensions: for participants who used multiplayer or socially interactive IVRE, questions addressed their experiences with competition, cooperation, and social connection. (4) Suggestions for improvement: participants were asked what features they would like to see improved or added to enhance their satisfaction with IVRE.

The interview protocol was pilot-tested with two IVRE users to ensure clarity and relevance of the questions, and minor adjustments were made based on their feedback.

#### 3.1.2 Interview implementation

All interviews were conducted by members of the research team who have received formal training in quantitative research methods and have practical experience in conducting semi-structured interviews in human-computer interaction research. To accommodate participants' preferences and geographical constraints, interviews were offered both in-person and online *via* video conferencing platforms. The choice of format was left to each participant.

Each interview session lasted between 45 and 75 min, with an average duration of approximately 55 min. All interviews were audio-recorded with the explicit consent of participants. Immediately following each session, the recordings were transcribed verbatim by the research team. To ensure accuracy, an author randomly selected 20% of the transcripts and compared them against the original audio recordings; no significant discrepancies were identified.

#### 3.1.3 Participant recruitment and characteristics

To ensure that the raw data addressed the research questions in a comprehensive, objective, and authentic manner, the interview design and procedures were informed by prior studies (Hu et al., 2023; Qi et al., 2024; Tian et al., 2022). The inclusion criteria for participants were as follows: (1) participants must have experience using multiple types of IVRE; (2) participants must demonstrate a clear understanding of the interview questions and be able to articulate their views effectively; and (3) participants must provide informed consent for participation and audio recording after being fully informed of the interview procedures and relevant regulations (Tian et al., 2022).

To adhere to the principle of diversity, participants were selected from different disciplinary backgrounds and age groups. Following the constant comparative method, responses to each interview question were continuously compared during data collection, and the sequence of questions was adjusted in real time as necessary. Theoretical saturation sampling was applied, whereby interview data were systematically organized, coded, and analyzed after each session. When no new categories emerged after cross-validation with at least three additional textual sources, theoretical saturation was considered achieved, and the data collected at this stage constituted the final sample (Hu et al., 2023). Ultimately, 16 participants meeting the inclusion criteria were selected for the study. The participants' basic characteristics are presented in Table 1.

**Table 1:** Participant information.

ID	Gender	Age	Education	Occupation	Usage frequency	Preferred IVRE
A1	Female	28	Undergraduate	Company employee	Occasionally	Beat Saber, Pistol Whip
A2	Male	25	Undergraduate	Teacher	Frequently	Eleven Table Tennis, Beat Saber
A3	Male	29	Postgraduate	Teacher	Frequently	Eleven Table Tennis, All-In-One Sports VR
A4	Female	26	Postgraduate	Student	Occasionally	Body Combat VR
A5	Male	24	Undergraduate	Company employee	Occasionally	Blade and Sorcer
A6	Male	25	Postgraduate	Student	Occasionally	All-In-One Sports VR
A7	Female	21	Undergraduate	Student	Frequently	All-In-One Sports VR, Beat Saber
A8	Female	28	Specialist	Freelancer	Occasionally	Body Combat VR
A9	Male	22	Undergraduate	Company employee	Occasionally	Pistol Whip, Beat Saber
A10	Male	29	Doctoral	Student	Frequently	The Thrill of the Fight, Pistol Whip
A11	Male	37	Doctoral	Teacher	Frequently	Beat Saber, All-In-One Sports VR
A12	Male	48	Postgraduate	Freelancer	Occasionally	GOLF+, Beat Saber
A13	Female	53	Doctoral	Teacher	Occasionally	GOLF+, All-In-One Sports VR
A14	Female	19	Specialist	Student	Frequently	Blade and Sorcery
A15	Male	20	Specialist	Company employee	Frequently	The Thrill of the Fight, Beat Saber
A16	Male	21	Undergraduate	Student	Occasionally	Blade and Sorcery, Pistol Whip

### 3.2 Open coding

Open coding represents the initial stage of conceptual refinement of raw interview data to delineate the scope of the research questions. At this stage, interview transcripts are examined line by line to identify core meanings and generate basic categories (Hu et al., 2023; Qi et al., 2024). These initial concepts are then systematically organized and grouped to form basic categories, enabling the structured classification of interview data (Goldkuhl and Cronholm, 2010; Hu et al., 2023; Tian et al., 2022). Through the open coding process, this study identified 57 basic categories. The detailed coding and categorization procedures for these basic categories are presented in Appendix A.

### 3.3 Axial coding

Axial coding builds upon open coding by exploring the logical relationships among categories and systematically integrating basic categories to clarify their interconnections, thereby generating higher-level abstract concepts (Hu et al., 2023; Qi et al., 2024; Tian et al., 2022; Yu et al., 2024; Zhao et al., 2024). During this phase, the basic categories identified through open coding were inductively refined and reorganized. As a result, fourteen basic categories were derived: Gamified experience design, System rewards and achievements, Challenge-oriented design, Personalized virtual configurations, Effective and health-oriented exercise, Appropriate and adaptive exercise guidance, Usage cost, Customizable system settings, Accessibility-oriented design, User-friendly interaction design, User-friendly interface design, Social interaction design, Competitive modes, and Cooperative modes. Detailed descriptions of these domains are provided in Table 2.

**Table 2:** The results of axial coding.

Main category	Subcategory	Basic categories
A1. Enjoyment	B1. Gamified experience design	C1. Game elements; C2. Gamified experiential quality; C3. Level design; C4. Pacing and flow
	B2. System rewards and achievements	C5. Points and leaderboards; C6. Engaging reward mechanisms
	B3. Challenge-oriented design	C7. Task challenges; C8. Level challenges; C9. Challenge events; C10. Competitive challenges; C11. Time-limited challenges
	B4. Personalized virtual configurations	C12. Avatar appearance; C13. Avatar equipment; C14. Avatar skills; C15. Personalized environment; C16. Socially supportive virtual presence
A2. Effectiveness	B5. Effective and health-oriented exercise	C17. Exercise effectiveness; C18. Health monitoring and feedback; C19. Appropriate exercise formats; C20. Injury risk reduction; C21. Mental health benefits
	B6. Appropriate and adaptive exercise guidance	C22. Adaptive to different fitness levels; C23. Adjustable exercise intensity; C24. Variety of exercise options; C25. Well-structured exercise plans; C26. Exercise instruction and guidance
A3. Usability	B7. Usage cost	C27. Perceived cost
	B8. Customizable system settings	C28. Ease of operation; C29. Adjustable size; C30. Customizable interface settings; C31. Customizable game content
	B9. Accessibility-oriented design	C32. Device compatibility; C33. Simplified device setup; C34. Accessibility; C35. Spatial flexibility; C36. Clear user manuals; C37. Video-based tutorials; C38. Human customer support
A4. Interactivity	B10. User-friendly interaction design	C39. Interaction flow; C40. Supportive and timely feedback; C41. Reduced cognitive load; C42. Fault tolerance; C43. Adjustable control methods
	B11. User-friendly interface design	C44. Easy-to-understand information cues; C45. Aesthetically pleasing interface design; C46. Clear and well-structured interface layout
A5. Sociality	B12. Social interaction design	C47. Virtual social experiences; C48. Social media sharing; C49. Well-designed social mechanisms; C50. Friend invitations and challenges; C51. Virtual social spaces; C52. Social activities and events; C53. Social network integration
	B13. Competitive modes	C54. Competitive activities; C55. Community-based competition
	B14. Cooperative modes	C56. Group-based cooperative activities; C57. Friend-based cooperation

### 3.4 Selective coding

Selective coding (Table 3) involves further synthesis and abstraction based on the results of open and axial coding. At this stage, data are systematically integrated and refined to elucidate the relationships between the main category and the USAT, thereby forming a coherent conceptual framework (Hu et al., 2023).

**Table 3:** Selective coding results.

Typical relationship structures	Influential relationship	Relationship structure connotation	Representative statements	References
A1 → USAT	Positive influence	IVREs that are highly enjoyable can elicit positive user emotions, increase engagement, reduce fatigue, and, consequently, improve USAT.	I think the traditional exercise methods are rather monotonous. However, through IVRE, engaging in physical activities offers me an interesting way to exercise.	(Krishnan et al., 2023; Meşe and Dursun, 2018; Rahmadiva et al., 2019)
A2 → USAT	Positive influence	An effective IVRE allows users to perceive its health benefits during and after use, thereby cultivating positive usage attitudes and ultimately enhancing USAT.	Using IVRE for exercise not only allows me to feel the effects of the workout, but also tracks my performance.	(Farič et al., 2019; Krishnan et al., 2023; Yao and Kim, 2019)
A3 → USAT	Positive influence	The usability of IVRE significantly affects the user experience journey, shaping users' perceptions of its value, sense of control, and trust, which in turn influences overall USAT.	IVRE makes it easier for me to learn how to use it, which has made my entire experience very good.	(Hassan, 2023; Rhiu et al., 2020; Stamm et al., 2020)
A4 → USAT	Positive influence	High-quality interactivity is a critical determinant of user engagement and experience, as positive interactive interactions directly enhance enjoyment during use and, in turn, increase USAT.	During initial use, I found myself relying more heavily on the software's beginner tutorials and instructions, and I would prefer the interaction methods to align with my everyday habits.	(Ahn et al., 2023; Stamm et al., 2020)
A5 → USAT	Positive regulatory effect	The social attributes of IVRE play a crucial role in satisfying users' social needs by enhancing their sense of engagement while facilitating communication and collaboration among players, which in turn strengthens user retention and ultimately increases overall USAT.	When exercising with friends, I find IVRE even more enjoyable. Not only does it provide a good workout, but it also strengthens the bonds between us.	(Li et al., 2020; Liszio et al., 2017; Maloney et al., 2021)

### 3.5 Testing for theoretical saturation

To ensure the robustness of the USAT evaluation indicators, three additional interview transcripts were analyzed after no new categories emerged during the coding process. The absence of new categories indicates that the theory derived from the coding has reached theoretical saturation (Francis et al., 2010; Turner and Astin, 2021). Based on this validation, the theoretical model developed in this study is considered to have achieved saturation.

### 3.6 Validation of the evaluation index system

To further assess the reliability and structural validity of the constructed USAT evaluation index system, we conducted a validation study following established psychometric practices. This validation utilized the questionnaire data collected from 226 valid respondents, which was also used for the CRITIC weighting analysis.

(1) Reliability analysis
  Internal consistency reliability was assessed using Cronbach's α coefficient. This coefficient measures the extent to which items within the same dimension consistently measure the same underlying construct. Reliability was calculated for each of the five first-level dimensions (Enjoyment, Effectiveness, Usability, Interactivity, Sociality) and for the overall scale. The analysis results show that the Cronbach's alpha values all reached above 0.70, indicating good internal consistency
(2) Exploratory factor analysis
  The results of the assessment of the applicability of the data show that the KMO value is 0.87, which is much higher than the recommended threshold of 0.70. This indicates that the sample size is sufficient. The Bartlett's sphericity test was significant (χ^2^ = 3842.56, df = 1596, *p* < 0.001), which confirmed that the correlation matrix was suitable for factor analysis. The EFA analysis extracted five factors with eigenvalues greater than 1, which collectively explained 68.4% of the total variance and the extracted factors were consistent with the theoretical hypotheses.

### 3.7 Framework for evaluation indicator system

**Figure 2:**
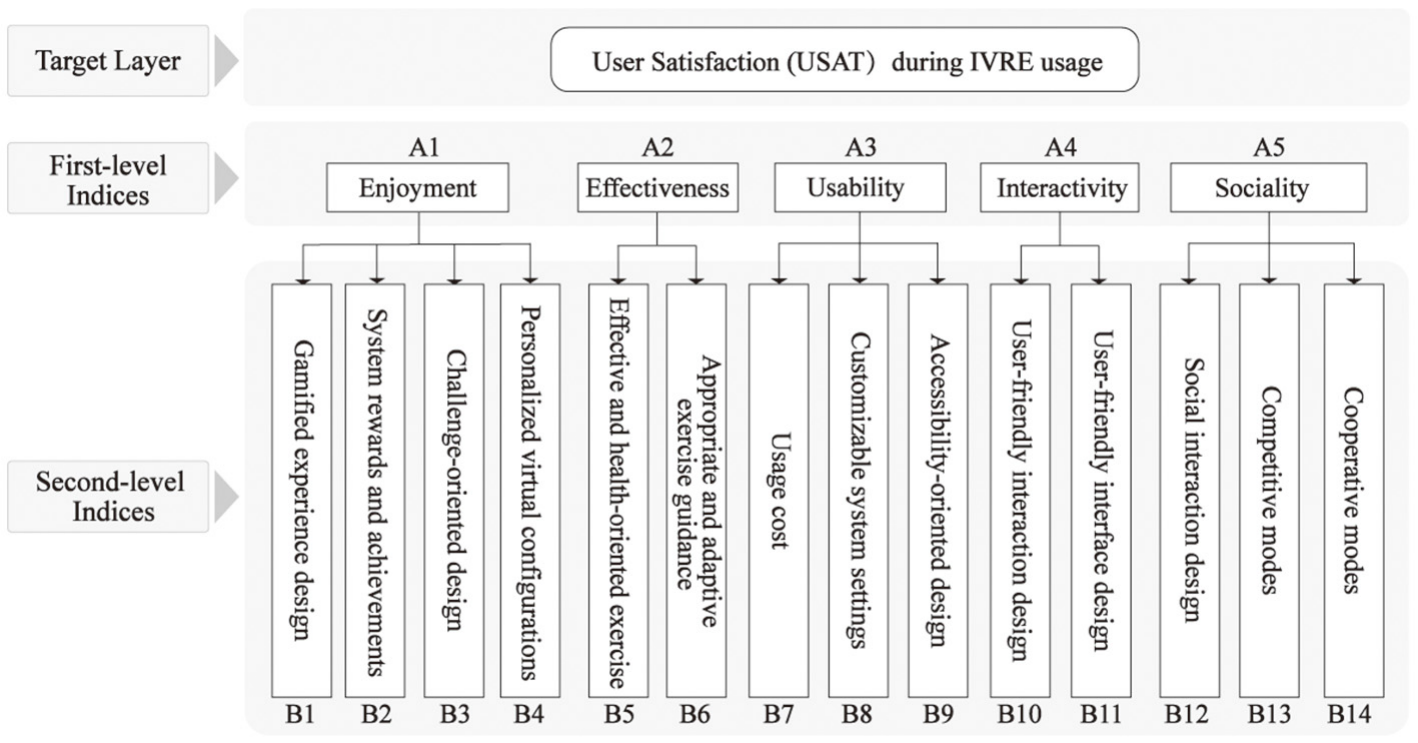
Framework of the USAT evaluation indicator system.

This study employed open coding, axial coding, and selective coding to generate three hierarchical coding levels. By systematically analyzing the relationships among these categories, a comprehensive framework for the USAT evaluation indicator system within the IVRE context was constructed, as illustrated in Figure 2.

## 4 Weighting indicators utilizing the G1-CRITIC method

This section utilizes the G1-CRITIC model to perform a quantitative analysis of the main categories, subcategories, and basic categories identified through grounded theory. Main categories are designated as first-level indicators in the evaluation metric system, subcategories as second-level indicators, and basic categories as third-level indicators.

### 4.1 Subjective weighting using the G1 method

The G1 method was applied to determine the subjective weights of the indicators within the evaluation framework, following a structured procedure, adhering to the following steps:

(1) Eight experts with extensive experience in IVRE research were invited to assess the relative importance of each indicator through interviews, based on the evaluation criteria presented in Table 4.
(2) Based on the experts' scoring results, the evaluation indicators are ranked in descending order of importance. The ordered sequence of indicators is denoted as *H*_1_,*H*_2_,*H*_3_,…, *H*_*n*_.
(3) The importance ratios between adjacent indicators are then calculated. For the *k* expert, the ratio of the importance scores assigned to two adjacent indicators is taken as the weighting ratio. The weighting ratio between indicators *H*_*k*−1_ and *H*_*k*_ is denoted as *R*_*k*_ and is calculated according to Equation 1.
$$\begin{array}{l} R_{k}=\frac{H_{k-1}}{H_{k}}(k=1,2,\ldots ,n) \end{array}$$ (1)
(4) The weighting factors for each indicator are then calculated, with the resulting indicator weights denoted as *R*_*k*_. The calculation of *R*_*k*_ is defined by Equation (2, the normalization of *W*_*k*_ is given in Equation 3, and the computation of the weight for *W*_*k*−1_ is presented in Equation 4.
  $$\begin{array}{l} R_{k}=\frac{H_{k-1}}{H_{k}}=\frac{W_{k-\;1}}{W_{k}} \end{array}$$ (2)
  $$\begin{array}{l} W_{k}=\frac{1}{1+\sum_{k=2}^{n}\left(\prod_{l=k}^{n}R_{l}\right)} \end{array}$$ (3)
  $$\begin{array}{l} W_{k-1}=W_{k}\cdot R_{k}=\frac{H_{k-1}}{H_{k}} \end{array}$$ (4)
(4) The subjective weights of the primary, secondary, and tertiary indicators in the USAT during IVRE usage evaluation framework were determined using the G1 method (Appendix B).

**Table 4:** Expert assessment criteria.

Importance level	Score
Highly important	9~10
Important	6~8
Moderately important	3~5
Not important	1~2

### 4.2 Objective weighting using the CRITIC method

The CRITIC method determines index weights by considering both the contrast intensity and the correlations among indices. In this study, eight representative IVRE products were selected, and questionnaires were administered to obtain scores for each evaluation index. A total of 250 questionnaires were distributed, yielding 226 valid responses. The sample consisted of 62.6% male and 37.4% female respondents and included participants from diverse occupational backgrounds, such as company employees, students, and teachers. The specific procedures of the CRITIC weighting method are described as follows:

(1) The correlation coefficient matrix is constructed based on the correlation coefficients, as defined in Equation 5, where *a*_*ij*_ represents the value of the *j* index for the *i*-th alternative.
$$\begin{array}{c} A=\left[a_{ij}\right]m*n \end{array}$$ (5)
(2) Dimensionless data processing: to ensure quantitative comparability among evaluation indices, standardization is applied to normalize all values to the range [0,1]. Indices with extremely small and extremely large characteristics are treated differently. Index A7 is classified as an extremely small index, for which lower values correspond to higher user satisfaction. All other indices are considered extremely large, with higher values indicating greater user satisfaction. The normalization methods for extremely large and extremely small indices are presented in Equations 6, 7, respectively.
  $$\begin{array}{c} X_{ij}=\frac{X_{ij}-Min\leq i\leq n\;X_{ij}}{Max\leq i\leq nX_{ij}-Min\;X_{i}\leq i\leq n\;X_{ij}} \end{array}$$ (6)
  $$\begin{array}{c} X_{ij}=\frac{Max\leq i\leq nX_{ij}-X_{ij}}{Max\leq i\leq nX_{ij}-Min\;X_{i}\leq i\leq n\;X_{ij}} \end{array}$$ (7)
(3) Contrast strength calculation: index variability reflects the extent to which users' evaluations of a given index differ across alternatives, with higher variability indicating greater divergence in user perceptions. The variability σ_*j*_ represents the information content of the *i*-th index and is calculated according to Equation 8.
$$\begin{array}{c} \sigma_{j}=\sqrt{\frac{\sum_{i=0}^{n}{\left(X_{ij}-X_{j}\right)}^{2}}{n-1}} \end{array}$$ (8)
(4) Conflictive calculation: index conflictiveness assesses the degree of contradiction between different indices. Higher conflictiveness increases decision-making complexity, as greater trade-offs are required to balance competing indices. Let *r*_*ij*_ denote the correlation coefficient between two indices, and let *S*_*j*_ represent the conflict magnitude of index *j*. The overall conflict value for each index is obtained by summation, with the calculation of *S*_*j*_ given in Equation 9.
$$\begin{array}{c} S_{j}=\overset{m}{\underset{j=1}{\sum }}\left(1-r_{ij}\right) \end{array}$$ (9)
(5) Information content calculation: *C*_*j*_ denotes the amount of information contained under condition *i*. A higher information content indicates that users provide more explicit comparisons, which is more conducive to determining relative weights. The calculation formula of *C*_*j*_ is given in Equation 10.
$$\begin{array}{c} C_{j}=\sigma_{j}\overset{m}{\underset{i=1}{\sum }}\left(1-r_{ij}\right) \end{array}$$ (10)
(6) Relative weight calculation: the weight of each index is determined based on its variability and the correlations among indices. The relative weight of index *j*, denoted as *W*_*j*_, is calculated using Equation 11.
$$\begin{array}{l} W_{j}=\frac{C_{j}}{\sum_{k=\;1}^{m}C_{j}} \end{array}$$ (11)
  Based on the final calculations, the objective weights of each index in the USAT evaluation metric system for IVRE usage were determined (Appendix C).

### 4.3 Combined weighting of the G1 and CRITIC methods

To overcome the limitations of relying on a single weighting approach, this study integrates the improved G1 method with the CRITIC method for index weighting. The weights obtained using the G1 method are denoted as *W*_*k*_, while those derived from the CRITIC method are denoted as *W*_*j*_. These two sets of weights are then combined through a multiplicative normalization approach to obtain the composite weight *W*, as defined in Equation 12. The final weighting results are presented in Table 5.


$$\begin{array}{c} W=\frac{W_{k}W_{j}}{\sum W_{k}W_{j}} \end{array}$$ (12)

**Table 5:** Weighting results for the combination.

First-level indices (main category)	Weight value (%)	Second-level indices (subcategory)	Weight value (%)	Third-level indices (basic categories)	Weight value (%)
A1	32.10	B1	10.21	C1	1.77
				C2	2.44
				C3	2.24
				C4	3.76
		B2	7.26	C5	3.77
				C6	3.49
		B3	7.98	C7	2.03
				C8	1.65
				C9	1.26
				C10	1.48
				C11	1.57
		B4	6.65	C12	1.75
				C13	1.41
				C14	1.03
				C15	1.01
				C16	1.44
A2	14.46	B5	7.55	C17	1.81
				C18	1.44
				C19	1.68
				C20	1.48
				C21	1.15
		B6	6.91	C22	1.50
				C23	1.53
				C24	2.07
				C25	0.95
				C26	0.86
A3	18.69	B7	6.17	C27	6.17
		B8	6.27	C28	1.43
				C29	1.38
				C30	1.71
				C31	1.74
		B9	6.24	C32	0.76
				C33	0.94
				C34	0.99
				C35	0.76
				C36	0.84
				C37	0.82
				C38	1.12
A4	15.18	B10	7.22	C39	1.80
				C40	1.34
				C41	1.06
				C42	1.45
				C43	1.58
		B11	7.96	C44	3.12
				C45	2.61
				C46	2.23
A5	19.60	B12	7.51	C47	1.31
				C48	0.93
				C49	1.29
				C50	1.21
				C51	0.82
				C52	1.03
				C53	0.93
		B13	6.65	C54	3.27
				C55	3.38
		B14	5.44	C56	2.36
				C57	3.08

## 5 Quantitative validation of the evaluation index system using the VIKOR method

VIKOR is a multi-attribute decision-making method used to rank and select alternatives by calculating group utility values, individual regret values, and compromise solutions (Qi et al., 2024; Tuskan and Basari, 2023). Its core principle is to identify the ideal and anti-ideal solutions among all alternatives. The final comprehensive ranking is determined by the distances of each alternative from these reference solutions, where the ideal solution exhibits the minimum distance and the anti-ideal solution the maximum distance. In this study, eight representative IVRE products were selected and evaluated using questionnaire data collected for the CRITIC method. The eight IVRE platforms selected for this study were chosen based on the following three criteria: (1) Market recognition and user base: platforms were selected according to their sales rankings, user ratings, and review counts on major virtual reality content distribution platforms (including Steam, Oculus Store, and Meta Quest Store), ensuring that the products presented on the selected platforms have demonstrated market acceptance; (2) Cross-platform compatibility: priority was given to IVREs that support multiple virtual reality hardware systems (e.g., Meta Quest and PC VR), thereby expanding the potential user base and reducing platform-specific biases; (3) Language availability: only IVREs with a Chinese-language version were selected to ensure that participants could fully understand the content. The specific calculation procedures are outlined as follows:

(1) Normalization processing. All index types are uniformly transformed into extreme-scale indices using the formula given in Equation 13. Here, S1 denotes GOLF+, S2 denotes Beat Saber, S3 denotes Eleven Table Tennis, S4 denotes Body Combat VR, S5 denotes Blade and Sorcery, S6 denotes Pistol Whip, S7 denotes The Thrill of the Fight, and S8 denotes All-In-One Sports VR. The normalized data are presented in Table 6.
$$\begin{array}{c} Max\left(x\right)-x \end{array}$$ (13)
(2) The matrix is subjected to dimensionless processing to eliminate the influence of differing index dimensions, with the calculation formula provided in Equation 14, where *n* denotes the evaluation alternatives, and *m* denotes the evaluation indices. The resulting calculation matrix is presented in Table 7, and the corresponding results are shown in Table 8.
$$\begin{array}{c} Z_{ij}=\frac{X_{ij}}{\sqrt{\sum_{i-1}^{n}X_{ij}^{2}}} \end{array}$$ (14)
(3) Determination of the group utility value and individual regret value. First, the positive and negative ideal solutions are determined. The positive ideal solution is composed of the maximum value of each column, while the negative ideal solution is composed of the minimum value of each column. Based on the ideal solutions, the group utility value *S*_*i*_ is calculated according to Equation 15, and the individual regret value *R*_*i*_ is calculated according to Equation 16. In these equations, *W* denotes the weight ratios of each main category obtained using the G1 and CRITIC methods, and *Z*_*ij*_ represents the normalized value of *X*_*ij*_ processed according to Equation 13.
  $$\begin{array}{c} \begin{array}{c} S_{i}=\sum_{j=1}^{m}\frac{W_{j}\left(Z_{j}^{+}-Z_{ij}\right)}{\left(Z_{j}^{+}-Z_{j}^{-}\right)} \end{array} \end{array}$$ (15)
  $$\begin{array}{c} R_{i}={Max}_{i}\left(\frac{W_{j}\left(Z_{j}^{+}-Z_{ij}\right)}{Z_{j}^{+}-Z_{j}^{-}}\right) \end{array}$$ (16)
(4) Calculation of the compromise value *Q*_*i*_. The parameter *v* is defined as the compromise coefficient, with a value range of [0,1]. In this study, the mean value is adopted, and thus *v* is set to 0.5, which accounts for both group utility and individual regret. The compromise value *Q*_*i*_ is calculated using Equation 17, where $S^{-}=\max S_{i}$, $S^{+}=\min S_{i}$, $R^{-}=\max R_{i}$, $R^{+}=\;\min R_{i}$.
$$\begin{array}{c} Q_{i}=\frac{v(S_{i}-S^{+})}{S^{-}-S^{+}}+\frac{\left(1-v)(R_{i}-R^{+}\right)}{R^{-}-R^{+}} \end{array}$$ (17)
(5) The USAT of the eight IVREs was evaluated using the three measures. *S*_*i*_, *R*_*i*_ and *Q*_*i*_. According to the ascending order of *Q*_*i*_, the ranking results are obtained as follows: S2, S1, S6, S5, S3, S8, S7, and S4. A smaller value of *Q*_*i*_ indicates a higher level of USAT, as shown in Table 9.
(6) Result validation. A solution is identified as the optimal alternative when the following two conditions are simultaneously satisfied.

**Table 6:** Normalization results.

Second-level indices	S1	S2	S3	S4	S5	S6	S7	S8
B1	7	9	7	6	9	9	7	9
B2	9	7	8	8	7	7	8	8
B3	7	8	6	8	9	8	9	6
B4	9	7	8	6	9	7	9	5
B5	8	9	7	9	5	9	9	8
B6	7	8	9	10	7	8	8	6
B7	1	1	0	2	4	2	7	4
B8	7	7	8	7	8	7	9	6
B9	7	9	8	8	7	8	7	7
B10	8	8	8	9	7	8	6	9
B11	8	8	7	7	8	8	6	8
B12	8	6	9	6	6	6	6	7
B13	9	7	9	7	8	7	8	8
B14	7	4	8	6	5	4	4	5

**Table 7:** Computational matrix for dimensionless processing.

*x* =	*x*_11_	*x*_12_	……	*x*_1m_
	*x*_21_	*x*_22_	……	*x*_2m_
	……	……	……	……
	*x*_n1_	*x*_n2_	……	*x*_nm_

**Table 8:** Results of dimensionless processing.

Second-level indices	S1	S2	S3	S4	S5	S6	S7	S8
B1	0.311	0.400	0.311	0.266	0.400	0.400	0.311	0.400
B2	0.409	0.318	0.364	0.364	0.318	0.318	0.364	0.364
B3	0.321	0.367	0.275	0.367	0.413	0.367	0.413	0.275
B4	0.417	0.324	0.371	0.278	0.417	0.324	0.417	0.232
B5	0.349	0.392	0.305	0.392	0.218	0.392	0.392	0.349
B6	0.311	0.355	0.400	0.444	0.311	0.355	0.355	0.266
B7	0.105	0.105	0.000	0.210	0.419	0.210	0.734	0.419
B8	0.333	0.333	0.381	0.333	0.381	0.333	0.429	0.286
B9	0.323	0.416	0.369	0.369	0.323	0.369	0.323	0.323
B10	0.357	0.357	0.357	0.401	0.312	0.357	0.268	0.401
B11	0.375	0.375	0.329	0.329	0.375	0.375	0.282	0.375
B12	0.414	0.310	0.465	0.310	0.310	0.310	0.310	0.362
B13	0.402	0.313	0.402	0.313	0.357	0.313	0.357	0.357
B14	0.445	0.255	0.509	0.382	0.318	0.255	0.255	0.318

**Table 9:** USAT ranking of eight IVREs.

Second-level indices	S1	S2	S3	S4	S5	S6	S7	S8
B1	0.10	0.00	0.10	0.14	0.00	0.00	0.10	0.00
B2	0.00	0.07	0.04	0.04	0.07	0.07	0.04	0.04
B3	0.0	0.03	0.09	0.03	0.00	0.03	0.00	0.09
B4	0.00	0.03	0.02	0.05	0.00	0.03	0.00	0.06
B5	0.02	0.00	0.04	0.00	0.08	0.00	0.00	0.02
B6	0.05	0.03	0.02	0.00	0.05	0.03	0.03	0.07
B7	0.04	0.04	0.05	0.04	0.02	0.04	0.00	0.02
B8	0.04	0.04	0.02	0.04	0.02	0.04	0.00	0.05
B9	0.05	0.00	0.03	0.03	0.05	0.03	0.05	0.05
B10	0.02	0.02	0.02	0.00	0.05	0.02	0.07	0.00
B11	0.00	0.00	0.04	0.04	0.00	0.00	0.09	0.00
B12	0.03	0.08	0.00	0.08	0.08	0.08	0.08	0.05
B13	0.00	0.06	0.00	0.06	0.03	0.06	0.03	0.03
B14	0.01	0.04	0.00	0.02	0.03	0.04	0.04	0.03
**S**_***i***_	0.41	0.44	0.45	0.55	0.48	0.46	0.52	0.51
**R**_***i***_	0.10	0.08	0.10	0.14	0.08	0.08	0.10	0.09
**Q**_***i***_	0.14	0.11	0.28	1.00	0.24	0.18	0.53	0.40
Ranking	2	1	5	8	4	3	7	6

Condition 1 (Acceptable advantage): $Q\left[X_{\left(1\right)}\right]-Q\left[X_{\left(2\right)}\right]\geq \frac{1}{m-1}$, where *X*_(1)_ denotes the best-ranked alternative according to the *Q*_*i*_ values, and *X*_(2)_ represents the second-best alternative.

Condition 2 (Acceptable stability): The alternative *X*_(1)_ must also be ranked as the best solution in either the *S*_*i*_ ranking or the *R*_*i*_ ranking. In these rankings, a smaller value indicates a better alternative.

If Condition 1 is satisfied but Condition 2 is not satisfied, the compromise solution is defined as {*X*_(1)_, *X*_(2)_}.

If Condition 1 is not satisfied but Condition 2 is satisfied, the compromise solution is defined as {*X*_(1)_, *X*_(2)_…, *X*_(*n*)_}, where *X*_(*n*)_ satisfies $Q\left[X_{\left(n\right)}\right]-Q\left[X_{\left(1\right)}\right]\leq \frac{1}{m-\;1}$.

**Figure 3:**
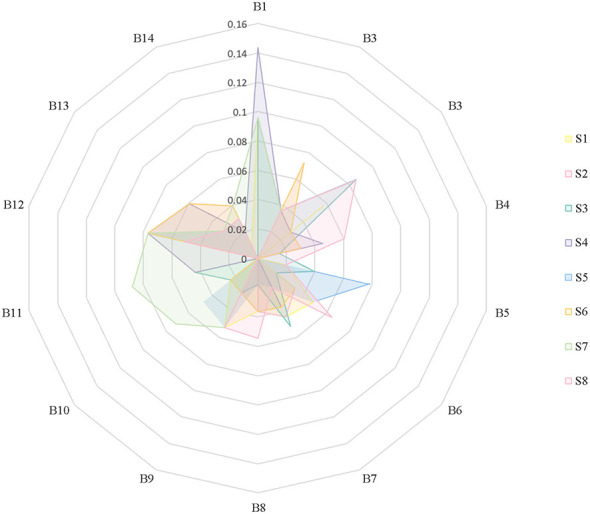
USAT results of second-level indices across eight IVREs.

Based on the validation results, S2 is identified as the IVRE with the highest USAT level among the eight alternatives. In addition, according to the user questionnaire evaluations, S2 also obtained the highest user rating among the eight IVREs. The consistency between the VIKOR evaluation results and the user-reported satisfaction ratings indicates that the USAT evaluation index system constructed in this study is effective for assessing USAT during IVRE usage (Figure 3).

## 6 Additional requirements establishment of the USAT evaluation index system for IVRE usage

**Figure 4:**
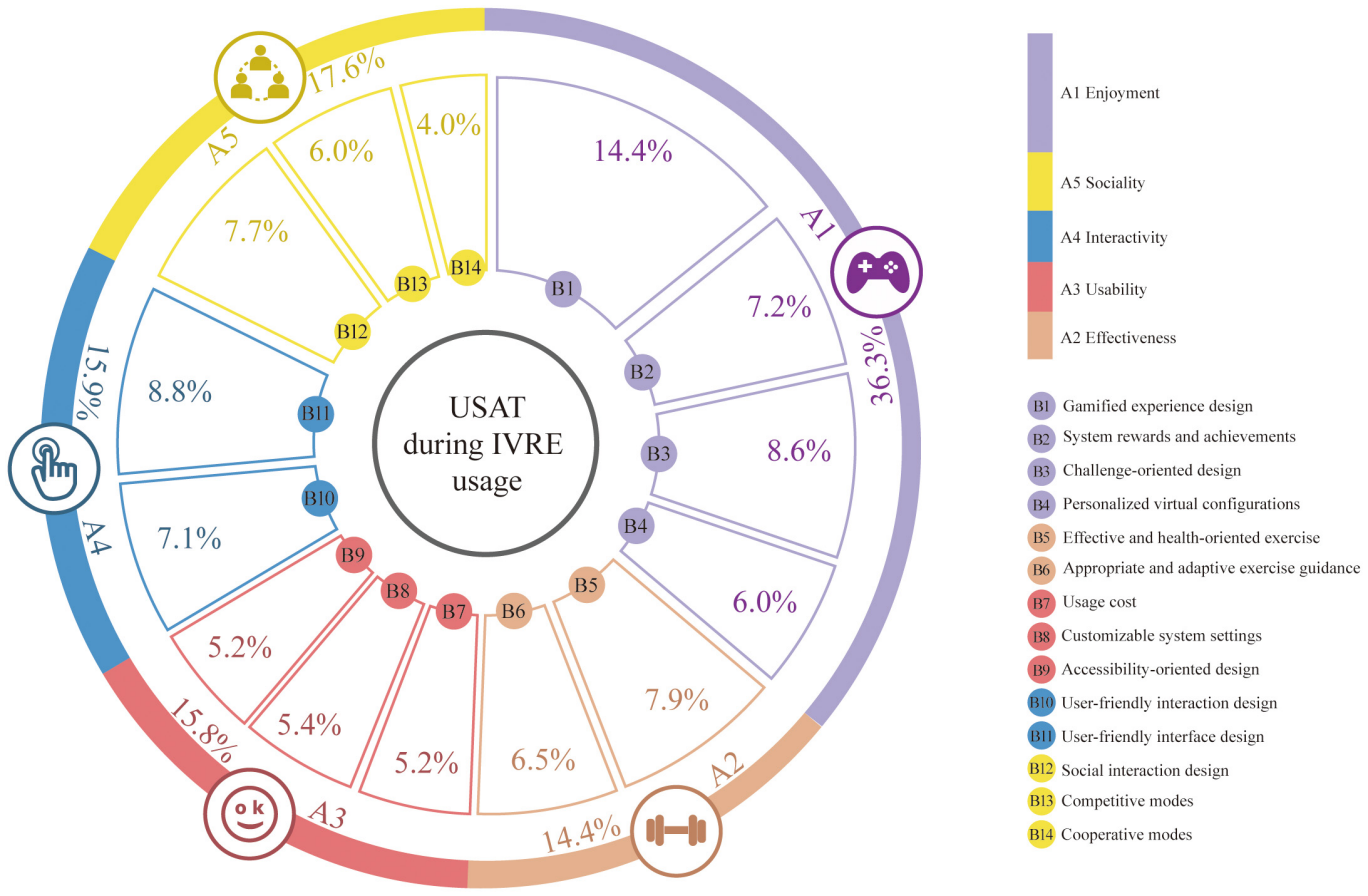
USAT evaluation index system during IVRE usage.

The results indicate that multiple factors, including enjoyment, effectiveness, usability, interactivity, and sociality, influence USAT during IVRE usage. Among these dimensions, enjoyment has the highest weight, followed by sociality, while interactivity, usability, and effectiveness rank in that order, as shown in Figure 4. The specific influencing factors and their respective degrees of impact are discussed in detail below.

Enjoyment denotes the pleasure and recreational value users experience during interaction with IVRE. By incorporating intuitive body-based motion controls, IVRE enhances gameplay vividness and immersion, thereby improving overall USAT (Krishnan et al., 2023; Meşe and Dursun, 2018; Rahmadiva et al., 2019). Among all influencing factors, enjoyment exerts the strongest positive effect on USAT, accounting for 36.3% of the explained variance. This dimension comprises four secondary indices: gamified experience design (B1), system rewards and achievements (B2), challenge-oriented design (B3), and personalized virtual configurations (B4). Among these, gamified experience design (B1) has the highest weight (14.4%), followed by challenge-oriented design (B3) and system rewards and achievements (B2).

Sociality refers to the capacity for interaction and social communication among users when engaging with IVRE. By incorporating appropriate social mechanisms, IVRE fosters a sense of belonging and social fulfillment during usage, thereby enhancing USAT (Li et al., 2020; Liszio et al., 2017; Maloney et al., 2021). Sociality ranks as the second most influential index affecting USAT, accounting for 17.6% of the variance and having a positive impact on USAT. This dimension comprises three second-level indices: social interaction design (B12, 7.7%), Competitive modes (B13, 6.0%), and Cooperative modes (B14, 4.0%).

Interactivity denotes the extent and quality of interaction between users and IVRE systems. Higher levels of interactivity enhance user engagement and involvement, thereby positively influencing USAT (Ahn et al., 2023; Stamm et al., 2020). Interactivity is the third most influential index affecting USAT during IVRE usage, accounting for 15.9% of the explained variance and exhibiting a positive relationship with USAT. This dimension comprises two second-level indices: User-friendly interface design (B11) and User-friendly interaction design (B10), with weights of 8.8 and 7.1%, respectively.

Usability refers to the degree to which users can easily learn, understand, and operate an IVRE system. High usability reduces users' learning costs and operational frustration, thereby enhancing overall USAT (Hassan, 2023; Rhiu et al., 2020; Stamm et al., 2020). Usability is the fourth most influential index affecting USAT, accounting for 15.8% of the variance and positively affecting user satisfaction. This dimension comprises three second-level indices: Customizable system settings (B8), Accessibility-oriented design (B9), and Usage cost (B7), each with a weight of 5.4, 5.2, and 5.2%, respectively.

Effectiveness refers to the extent to which IVRE achieves its intended functional objectives, such as fitness enhancement, rehabilitation training, education, and skill development (Farič et al., 2019; Krishnan et al., 2023; Yao and Kim, 2019). It is a key characteristic that differentiates IVRE from conventional digital games. When users perceive that IVRE can effectively meet their personal goals and needs, their level of USAT increases accordingly. Effectiveness is the fifth major index influencing USAT, accounting for 14.4% of the variance, and it has a positive impact on USAT. This dimension comprises two secondary indices: Effective and health-oriented exercise (7.9%) and Appropriate and adaptive exercise guidance (6.5%).

## 7 Discussion and conclusion

### 7.1 Discussion

Among the first-level indices, enjoyment accounts for the largest proportion, indicating that it is the core index influencing USAT. This finding strongly aligns with Flow Theory (Csikszentmihalyi, 1990). The high weight of sub-indices such as “pacing and flow” and “challenge-oriented design” empirically confirms that users achieve optimal experience when they are immersed in an activity with clear goals and immediate feedback, where their skills are commensurate with the challenge. Enjoyment, in this context, is not merely a fleeting emotion but the result of achieving a flow state, which intrinsically motivates users to continue their exercise regimen (Meşe and Dursun, 2018; Rahmadiva et al., 2019). This motivational role of enjoyment has been consistently observed across various populations and contexts. For instance, immersive VR environments have been proven to enhance the enjoyment and motivation of exercisers, thereby increasing their engagement and improving their physical health (John et al., 2025; Lai et al., 2020; Lemmens, 2023). Therefore, to improve USAT, IVRE systems should prioritize the enhancement of these gamified experiential aspects by integrating richer gamification elements to evoke positive emotional responses (Meşe and Dursun, 2018), incorporating appropriate levels of challenge to avoid boredom or frustration (Lee et al., 2021; Sweetser and Wyeth, 2005), and establishing sustainable reward and achievement systems (Gary et al., 2017). Furthermore, personalized virtual settings—such as avatar customization and environment personalization—can enhance users' sense of autonomy and immersion, thereby supporting long-term user retention (Poncin and Garnier, 2012).

Sociality also plays a crucial role in enhancing USAT during IVRE use, exerting a substantial positive influence. This can be robustly explained by Self-Determination Theory (Ryan and Deci, 2000). The positive impact of social interaction design, competitive modes and cooperative modes directly addresses the psychological need for relatedness. By satisfying this innate need for belonging and social recognition through interpersonal interaction (Liszio et al., 2017; Maloney et al., 2021), IVRE transforms exercise from a solitary chore into a socially rewarding activity. This fulfillment of relatedness not only strengthens users' engagement and retention but also stimulates exercise motivation (Shah et al., 2023). To fully leverage this, IVRE systems should diversify social interaction formats by providing virtual social spaces for interaction with unfamiliar users and implementing invitation mechanisms to encourage engagement with acquaintances. Offering a range of competitive modes and establishing effective collaborative mechanisms through shared goals and cooperative tasks can further stimulate user participation, enhance group cohesion, reduce users' sense of isolation, and reinforce their sense of belonging (Li et al., 2020).

Customisable system settings, accessibility-oriented design, and usage cost constitute three second-level indices that significantly influence user experience during IVRE interaction and play a critical role in shaping USAT. Usability helps reduce users' cognitive load and lowers participation barriers, thereby broadening the potential user base of IVRE (Xu et al., 2023). Consequently, IVRE design should prioritize usability by offering personalized adjustment options across both software and hardware dimensions to accommodate diverse user needs. This design process must place strong emphasis on accessibility, taking into account the heterogeneous capabilities and requirements of different user groups (Brooks, 2017). Complementing these design efforts, adopting reasonable pricing strategies and providing effective post-deployment maintenance and support are essential to encourage wider adoption and sustained user participation (Farič et al., 2019).

Interactivity constitutes one of the first-level indices within the USAT evaluation index system for IVRE usage. Strong interactivity not only enhances user immersion but also facilitates emotional resonance, thereby contributing to positive user experiences (Ahn et al., 2023). This finding is consistent with a broad body of qualitative and user-experience research, which has demonstrated that higher levels of interactivity consistently increase user engagement and perceived quality of experience (Davidavičiene et al., 2021; Deng et al., 2025; Kim and Ha, 2021) The interactivity of IVRE can be strengthened by establishing user-friendly human–computer interaction methods, including simple, intuitive operational workflows, responsive feedback mechanisms, and high fault tolerance (Su et al., 2020). This should be complemented by a user-friendly interface design, featuring aesthetically appealing and clearly structured interfaces that support efficient interaction and reduce user effort (Kim and Ha, 2021; Su et al., 2020).

Effective and health-oriented exercise and Appropriate and adaptive exercise guidance constitute two second-level indices that influence users' perceptions of effectiveness when using IVRE, thereby exerting a significant impact on USAT. Effectiveness refers to the extent to which users achieve their anticipated exercise outcomes through IVRE usage. Therefore, the design and development of IVRE must ensure the appropriateness of exercise intensity and the effectiveness of exercise outcomes by delivering demonstrably effective workout results and adopting suitable exercise formats (Farič et al., 2019). In addition, IVRE should provide scientifically grounded, adaptive exercise guidance to reduce injury risk during physical activity and support safe, sustainable user engagement.

### 7.2 Conclusion

This study first employed grounded theory to develop an evaluation index system for USAT during IVRE use. The first-level indices comprise five dimensions: enjoyment, effectiveness, usability, interactivity, and sociality. The second-level indices consist of fourteen dimensions, while the third-level indices encompass fifty-seven dimensions. Subsequently, the G1–CRITIC combined weighting method, integrating both subjective and objective weighting approaches, was applied to determine the weight for each index. The results indicate that enjoyment, sociality, and usability exhibit relatively higher weights among the first-level indices. At the second level, indices such as gamified experience design, user-friendly interface design, challenge-oriented design, effective and health-oriented exercise, and social interaction design demonstrate particularly prominent weightings. At the third level, indices including perceived cost, points and leaderboards, pacing and flow, and engaging reward mechanisms carry significant weights. Finally, the proposed evaluation index system was quantitatively validated using the VIKOR method across eight popular IVREs currently available in the market. The results confirm the high validity of the constructed system. This evaluation index system enables IVRE enterprises to identify key indices for enhancing USAT and systematically review and address existing shortcomings. It provides theoretical support for developers during the design and development phases.

## 8 Limitations and future research

Despite its contributions, this study has several limitations that warrant acknowledgment. First, the sample used for qualitative data collection was demographically restricted, and the quantitative validation sample was derived from a convenience sample. As a result, the findings may not fully capture the needs of older adults, children, or users with varying educational backgrounds. Future research should employ stratified sampling to improve generalizability. Second, the cross-sectional design captures user satisfaction at a single time point, although satisfaction is dynamic and may evolve with increased familiarity and shifting user goals. Longitudinal studies that track satisfaction across multiple time points would help elucidate the factors driving initial adoption versus long-term retention. Third, the study did not differentiate across IVRE genres beyond platform-based classification, even though different genres—such as rhythm-based games vs. sports simulations—may prioritize distinct satisfaction factors. Future research should conduct comparative analyses across genres to develop more tailored evaluation frameworks. Fourth, the study was conducted within specific cultural and technological contexts. Cultural factors and the rapid pace of VR advancement may influence satisfaction in ways that limit the generalizability of the findings to other contexts or future generations of technology. In light of these limitations, the conclusions should be interpreted as providing preliminary evidence for the proposed evaluation framework, rather than as definitive or universally applicable claims. The framework offers a theoretically grounded starting point, and future research is needed to validate, refine, and extend it across diverse populations, longitudinal designs, and evolving technological contexts.

## Data Availability

The original contributions presented in the study are included in the article/Supplementary material, further inquiries can be directed to the corresponding author/s.
