# Correction: Construction of an evaluation index system for user satisfaction with immersive virtual reality exergaming

**DOI:** 10.3389/fpsyg.2026.1862646

**Published:** 2026-05-19

**Authors:** Jian Fu, Nanyue Mou, Duoyou Gong, Youzhi Xu, Xiaoning Lu

**Affiliations:** 1 College of Physical Education and Health, Guangxi Normal University, Guilin, Guangxi, China; 2 Guangxi College of Sports Education, Nanning, Guangxi, China; 3 Guilin University, Guilin, Guangxi, China; 4 Tongmyong University, Busan, Republic of Korea; 5 Silla University, Busan, Republic of Korea

**Keywords:** evaluation index system, G1-CRITIC, grounded theory, immersive virtual reality exergaming, user satisfaction, VIKOR

Affiliation 3 Guilin University, Guilin, Guangxi, China was erroneously given as 3 School of Tourism, Physical Education and Health, Guangxi Normal University, Guilin, Guangxi, China.

The original version of this article has been updated.

