# Supplementary material for: Construction of an evaluation index system for user satisfaction with immersive virtual reality exergaming
Source: Front Psychol. 2026 Apr 17;17:1811648. doi: 10.3389/fpsyg.2026.1811648 (PMC13134464; doi:10.3389/fpsyg.2026.1811648)
Supplement: Supplementary file 1 [file Supplementary_file_1.docx]

Supplementary Material

# Appendix A Open coding process.

| **Representative statement information** | **Conceptualization** | **Basic categories** |
| --- | --- | --- |
| It doesn’t feel like simple exercise—the game elements make it engaging and fun | Many game-like elements | Game elements |
| The overall experience feels very much like playing a real game rather than just working out. | An exercise experience like playing games | Gamified experiential quality |
| Each level is well designed, and progressing through them keeps me motivated to continue. | Interesting and appropriate level design | Level design |
| The pace of the game feels just right, and I can easily get into a smooth rhythm while playing. | A well-paced rhythm | Pacing and flow |
| I particularly enjoy features like leaderboards that showcase my prowess—they stoke my competitive spirit and make me want to play even more. | Points, rankings and other honours | Points and leaderboards |
| The points system is brilliant; completing tasks or challenges earns me points, and watching my tally grow bit by bit gives me a real sense of achievement. |  |  |
| Every time I finish a task, I eagerly anticipate what special reward I'll receive. | The reward system serves as an incentive | Engaging reward mechanisms |
| Rewards act as motivation—sometimes I'll complete tasks just to get a particular one. |  |  |
| New challenge tasks are truly thrilling moments—I'm always eager to accept them. | Challenging tasks prove exhilarating | Task challenges |
| Its level design is excellent, offering a satisfying challenge while igniting my competitive spirit. | Level design engages users emotionally | Level challenges |
| I find immense joy in clearing each stage and eagerly anticipate the next. When discussing progress with friends, I take pride in having conquered numerous levels. |  |  |
| The daily login challenges are delightful little activities; checking in each day has become a habit. | Daily challenges become a habit | Challenge events |
| I participate in every competition event, occasionally securing prizes through good fortune. | Competitions yield rewarding outcomes | Competitive challenges |
| I've met many interesting, like-minded people through competitions, and chatting with them is a real pleasure. |  |  |
| Special holiday events carry a sense of ceremony; celebrating in this way is quite enjoyable. | Unique and highly appealing festive activities | Time-limited challenges |
| Limited-time festive activities often contain hidden surprises, like receiving gifts. |  |  |
| My virtual avatar is just like me; I adore my character's appearance. | Virtual avatars as personalised tags | Avatar appearance |
| I love customising my avatar, feeling as though I'm truly present within the game. |  |  |
| Different equipment possesses distinct functions, so I focus on collecting gear I favour. | Collection and utilisation of functional tools | Avatar equipment |
| My gear symbolises my strength and capabilities... |  |  |
| My avatar's skills represent my unique abilities, making gameplay feel distinct from others. | Demonstration of unique skills | Avatar skills |
| During workouts, I select my preferred virtual environments to train in. | Selectable virtual environments | Personalized environment |
| I relish the cheers from the crowd, even knowing they're simulated. | Virtual audiences to stimulate positive emotions | Socially supportive virtual presence |
| With virtual spectators present, it feels as though I've truly entered an arena. |  |  |
| After playing for a while, I've genuinely noticed weight loss – it's genuinely effective. | Noticing real physical improvements | Exercise effectiveness |
| I'll monitor whether this game genuinely aids physical conditioning. |  |  |
| Tracking my calorie expenditure provides significant motivation for weight loss. | Tracking body data motivates exercise | Health monitoring and feedback |
| After each session, I meticulously review my workout metrics to assess my exertion level. |  |  |
| My primary focus remains finding exercises suited to my needs. | Discovering suitable exercise formats | Appropriate exercise formats |
| Once, after a workout, my muscles ached so badly I suspected a strain. | Professional exercise guidance | Injury risk reduction |
| Without professional coaching, I wouldn't attempt new exercise forms. |  |  |
| Each session releases work stress, leaving me feeling genuinely happy. | Alleviate daily stress | Mental health benefits |
| After playing for a while, I notice my mood has lifted. | Improve mood |  |
| For beginners like me, it's very beginner-friendly, not ramping up difficulty immediately. | Suitable for users of all fitness levels | Adaptive to different fitness levels |
| It's quite challenging, more so than other games. |  |  |
| When tired, I opt for the relaxed mode; when feeling energetic, I choose the challenging one. | Offers a range of exercise intensities | Adjustable exercise intensity |
| Downloading one game allows you to experience various sports, offering excellent value for money. | Experience diverse workout options | Variety of exercise options |
| Even without access to professional facilities, you can still experience different sports within the game. |  |  |
| When exercising alone, I'm unsure if my workout plan is reasonable. | Professional exercise programme design | Well-structured exercise plans |
| Having a structured plan makes it easier for me to stick to my routine. |  |  |
| I'd trust plans devised by professional trainers more readily. | Providing proper exercise guidance | Exercise instruction and guidance |
| When exercising alone, I'm unsure if my form is correct or what the proper technique should be. |  |  |
| I've been keen to try XX game, but its price has always deterred me. | Price feels too high | Perceived cost |
| The controllers feel comfortable to use, though I'm constantly worried about tripping over the cables. | Hardware equipment offering comfortable usage | Ease of operation |
| XX brand controllers feel more comfortable than XX brand ones. |  |  |
| My head is rather small, so I always feel the VR headset might slip off. | Appropriate dimensions | Adjustable size |
| I enjoy customising colours and layouts to tailor the interface to my preferences. | Interface configuration aligned with operational habits | Customizable interface settings |
| I always select game content I enjoy. | Aesthetically pleasing game content selection | Customizable game content |
| When choosing scenarios, I tend towards futuristic, tech-inspired themes. |  |  |
| My XX brand VR device is incompatible with XXX game, which is a real shame. | Compatible across various devices | Device compatibility |
| First-time setup always involves fiddly adjustments and learning controls. | User-friendly for first-time users | Simplified device setup |
| There are no accessible games for disabled users yet, meaning many can't participate. | Accessible to individuals with disabilities | Accessibility |
| Being able to play at home is genuinely convenient. | Minimal spatial constraints | Spatial flexibility |
| With no time for dedicated venues, IVRE serves as a practical alternative. |  |  |
| When encountering difficulties, I consult the user manual. | Requires user manual guidance | Clear user manuals |
| For issues, I search for solutions on XXX video platforms. | Requires video instruction | Video-based tutorials |
| For after-sales or system problems, I prefer contacting human customer support. | Requires human customer service | Human customer support |
| Complex interaction methods feel challenging to learn. | Simple interaction methods | interaction flow |
| Its interface mirrors what I've used before, requiring little effort. |  |  |
| I pressed it several times with no response, which was genuinely frustrating. | Provides timely feedback to users | Supportive and timely feedback |
| When the system malfunctions and doesn't respond to any input, my experience suffers. |  |  |
| I find excessive interface information irritating. | Moderate interface information | Reduced cognitive load |
| Its interface is straightforward, allowing me to focus well on the game. |  |  |
| Sometimes pressing the wrong button causes it to exit immediately, making me feel rather daft. | Allows quick reversal of erroneous actions | Fault tolerance |
| Occasionally I tap the wrong option, but thankfully it prompts for confirmation, so I quickly cancel. |  |  |
| Whenever I start a new game, I always configure it to my preferred playstyle. It's a bit of a faff, but it makes the experience far more comfortable. | Adjust to a control scheme that suits your habits | Adjustable control methods |
| I have external peripherals, so I customise the IVRE's control settings accordingly. |  |  |
| Key information is highlighted with clear prompts. | Provides clear prompts | Easy-to-understand information cues |
| Too much text-heavy guidance just obscures the essentials. |  |  |
| I'm rather fond of XX game's interface and have recommended it to friends. | Aesthetically pleasing interface | Aesthetically pleasing interface design |
| XX's colour scheme perfectly matches how I perceive this sport. |  |  |
| In XX game, I couldn't locate the settings section; the navigation was unclear. | Clear and easy-to-read interface, free from clutter | Clear and well-structured interface layout |
| The labels and icons are designed clearly, allowing me to quickly identify the functions they represent. |  |  |
| With my busy work schedule, I rarely meet new people. It's wonderful to connect with like-minded users in this IVRE community. | Enables you to connect with like-minded online friends | Virtual social experiences |
| I'll share my achievements with friends to show off a bit. | Share with friends | Social media sharing |
| I can hide information I don't wish to disclose publicly – this feature is very sensible. | Social privacy protection | Well-designed social mechanisms |
| I can block users I'd rather not interact with. | Social safeguards |  |
| I particularly enjoy the social features – chatting, exercising together, and tackling challenges with friends has deepened our bonds. | Interact with friends | Friend invitations and challenges |
| The community is full of like-minded individuals; exchanging ideas is genuinely enjoyable. | Provide virtual communities and gatherings | Virtual social spaces |
| The recent event was brilliant fun. I met a new user there, and we've stayed in touch ever since. | Offer opportunities to connect with online acquaintances | Social activities and events |
| Being able to easily find friends from XX's social platform within the game strengthens our connections. | Synchronise online and offline friends | Social network integration |
| XX Game's cross-platform friend invitations are utterly convenient. |  |  |
| The game regularly hosts competitive events; the intense rivalry truly captures the essence of sport. | Competitive activities present greater challenges | Competitive activities |
| My community features an Honours Wall – once I made it onto the list, it was sheer delight, a genuine highlight of my life. | Community contests encourage participation | Community-based competition |
| Every team match feels like fighting shoulder to shoulder. | Training fosters teamwork skills | Group-based cooperative activities |
| Competing alongside friends deepens our bonds beyond the games themselves. | Bringing friends closer together | Friend-based cooperation |

# Appendix B The subjective weight results of the G1 method.

| **First-level indices (Main Category)** | **Weight value** | **Second-level indices (Subcategory)** | **Weight value** | **Third-level indices (Basic categories)** | **Weight value** |
| --- | --- | --- | --- | --- | --- |
| A1 | 31.23% | B1 | 10.75% | C1 | 1.90% |
|  |  |  |  | C2 | 2.66% |
|  |  |  |  | C3 | 2.45% |
|  |  |  |  | C4 | 3.74% |
|  |  | B2 | 7.85% | C5 | 3.53% |
|  |  |  |  | C6 | 4.31% |
|  |  | B3 | 6.68% | C7 | 1.85% |
|  |  |  |  | C8 | 1.50% |
|  |  |  |  | C9 | 1.00% |
|  |  |  |  | C10 | 0.84% |
|  |  |  |  | C11 | 1.49% |
|  |  | B4 | 5.96% | C12 | 1.79% |
|  |  |  |  | C13 | 1.34% |
|  |  |  |  | C14 | 0.89% |
|  |  |  |  | C15 | 0.74% |
|  |  |  |  | C16 | 1.19% |
| A2 | 15.65% | B5 | 5.96% | C17 | 2.49% |
|  |  |  |  | C18 | 1.14% |
|  |  |  |  | C19 | 1.72% |
|  |  |  |  | C20 | 1.42% |
|  |  |  |  | C21 | 0.96% |
|  |  | B6 | 7.92% | C22 | 1.96% |
|  |  |  |  | C23 | 2.20% |
|  |  |  |  | C24 | 1.55% |
|  |  |  |  | C25 | 1.21% |
|  |  |  |  | C26 | 1.00% |
| A3 | 18.44% | B7 | 6.96% | C27 | 6.96% |
|  |  | B8 | 6.13% | C28 | 1.52% |
|  |  |  |  | C29 | 1.38% |
|  |  |  |  | C30 | 1.40% |
|  |  |  |  | C31 | 1.83% |
|  |  | B9 | 5.34% | C32 | 0.66% |
|  |  |  |  | C33 | 0.95% |
|  |  |  |  | C34 | 0.81% |
|  |  |  |  | C35 | 0.53% |
|  |  |  |  | C36 | 0.53% |
|  |  |  |  | C37 | 0.66% |
|  |  |  |  | C38 | 1.20% |
| A4 | 16.07% | B10 | 7.91% | C39 | 1.76% |
|  |  |  |  | C40 | 1.78% |
|  |  |  |  | C41 | 0.99% |
|  |  |  |  | C42 | 1.99% |
|  |  |  |  | C43 | 1.38% |
|  |  | B11 | 8.17% | C44 | 3.04% |
|  |  |  |  | C45 | 2.24% |
|  |  |  |  | C46 | 2.88% |
| A5 | 18.62% | B12 | 8.44% | C47 | 1.05% |
|  |  |  |  | C48 | 1.24% |
|  |  |  |  | C49 | 1.69% |
|  |  |  |  | C50 | 1.69% |
|  |  |  |  | C51 | 0.84% |
|  |  |  |  | C52 | 1.08% |
|  |  |  |  | C53 | 0.84% |
|  |  | B13 | 5.62% | C54 | 3.10% |
|  |  |  |  | C55 | 2.52% |
|  |  | B14 | 4.56% | C56 | 1.86% |
|  |  |  |  | C57 | 2.69% |

# Appendix C The subjective weight results of the G1 method.

| **First-level indices (Main Category)** | **Weight value** | **Second-level indices (Subcategory)** | **Weight value** | **Third-level indices (Basic categories)** | **Weight value** |
| --- | --- | --- | --- | --- | --- |
| A1 | 32.97% | B1 | 9.67% | C1 | 1.64% |
|  |  |  |  | C2 | 2.22% |
|  |  |  |  | C3 | 2.04% |
|  |  |  |  | C4 | 3.78% |
|  |  | B2 | 6.67% | C5 | 4.00% |
|  |  |  |  | C6 | 2.67% |
|  |  | B3 | 9.28% | C7 | 2.22% |
|  |  |  |  | C8 | 1.79% |
|  |  |  |  | C9 | 1.51% |
|  |  |  |  | C10 | 2.12% |
|  |  |  |  | C11 | 1.64% |
|  |  | B4 | 7.34% | C12 | 1.72% |
|  |  |  |  | C13 | 1.48% |
|  |  |  |  | C14 | 1.18% |
|  |  |  |  | C15 | 1.27% |
|  |  |  |  | C16 | 1.69% |
| A2 | 13.28% | B5 | 7.38% | C17 | 1.12% |
|  |  |  |  | C18 | 1.75% |
|  |  |  |  | C19 | 1.64% |
|  |  |  |  | C20 | 1.54% |
|  |  |  |  | C21 | 1.34% |
|  |  | B6 | 5.91% | C22 | 1.04% |
|  |  |  |  | C23 | 0.86% |
|  |  |  |  | C24 | 2.59% |
|  |  |  |  | C25 | 0.70% |
|  |  |  |  | C26 | 0.72% |
| A3 | 18.94% | B7 | 5.37% | C27 | 5.37% |
|  |  | B8 | 6.42% | C28 | 1.35% |
|  |  |  |  | C29 | 1.38% |
|  |  |  |  | C30 | 2.03% |
|  |  |  |  | C31 | 1.65% |
|  |  | B9 | 7.15% | C32 | 0.86% |
|  |  |  |  | C33 | 0.94% |
|  |  |  |  | C34 | 1.18% |
|  |  |  |  | C35 | 0.99% |
|  |  |  |  | C36 | 1.15% |
|  |  |  |  | C37 | 0.99% |
|  |  |  |  | C38 | 1.04% |
| A4 | 14.29% | B10 | 6.54% | C39 | 1.83% |
|  |  |  |  | C40 | 0.90% |
|  |  |  |  | C41 | 1.12% |
|  |  |  |  | C42 | 0.90% |
|  |  |  |  | C43 | 1.79% |
|  |  | B11 | 7.75% | C44 | 3.20% |
|  |  |  |  | C45 | 2.98% |
|  |  |  |  | C46 | 1.58% |
| A5 | 20.58% | B12 | 6.59% | C47 | 1.56% |
|  |  |  |  | C48 | 0.62% |
|  |  |  |  | C49 | 0.89% |
|  |  |  |  | C50 | 0.74% |
|  |  |  |  | C51 | 0.81% |
|  |  |  |  | C52 | 0.97% |
|  |  |  |  | C53 | 1.01% |
|  |  | B13 | 7.68% | C54 | 3.44% |
|  |  |  |  | C55 | 4.24% |
|  |  | B14 | 6.32% | C56 | 2.86% |
|  |  |  |  | C57 | 3.46% |
